# Family Caregiving and Depression among Older Adults in Japan: A Cross-Sectional Study during the COVID-19 Pandemic

**DOI:** 10.1093/geroni/igab046.2960

**Published:** 2021-12-17

**Authors:** Taiji Noguchi, Takahiro Hayashi, Yuta Kubo, Naoki Tomiyama, Akira Ochi, Hiroyuki Hayashi

**Affiliations:** 1 National Center for Geriatrics and Gerontology, Obu, Aichi, Japan; 2 Seijoh University, Tokai, Aichi, Japan

## Abstract

COVID-19 infections are particularly lethal in older adults; thus, social activities of older adults and their families in the community have been restricted. The threat of infection, restrictions on social activities, and limitations on the provision of care services for older adults could increase family caregivers’ burden and impact their mental health. This cross-sectional study examined the association between family caregiving and change in depression during the COVID-19 pandemic. In October 2020, we conducted a mailed questionnaire survey on a random sample of functionally independent community-dwelling older adults in a semi-urban area of Japan. Based on the depression status between March and October 2020, participants were classified into four groups: “consistently non-depressed,” “depression onset,” “recovering from depression,” and “remained depressed.” Participants were assessed for providing care for their family members or not. Caregiver participants were also assessed on their caregiving role (primary or secondary), the severity of their care-recipient’s needs, and an increased caregiver burden. Data from 957 older adults were analyzed. The participants’ mean age (SD) was 80.8 (4.8) years, and 53.5% were female. Multivariable multinomial logistic regression analysis revealed that family caregiving was associated with depression onset (OR=3.17 [95%CI=1.57-6.40], p=0.001) and remaining depressed (2.53 [1.36-4.71], p=0.004). Particularly, primary caregivers, those providing care for family members with severer care need-levels, and those with an increased caregiver burden had a higher risk of depression onset and remaining depressed. Family caregivers could have severe mental health conditions during the pandemic. Developing a support system is essential to protect their mental health.

